# Measurement of Rayleigh Wave Beams Using Angle Beam Wedge Transducers as the Transmitter and Receiver with Consideration of Beam Spreading

**DOI:** 10.3390/s17061449

**Published:** 2017-06-20

**Authors:** Shuzeng Zhang, Xiongbing Li, Hyunjo Jeong

**Affiliations:** 1 School of Traffic and Transportation Engineering, Central South University, Changsha 410075, China; zhangshuzeng123@163.com; 2 Division of Mechanical and Automotive Engineering, Wonkwang University, Iksan 570-749, Korea; hjjeong@wku.ac.kr

**Keywords:** Rayleigh wave, angle beam wedge transducer, leaky Rayleigh wave theory, area integral model

## Abstract

A theoretical model, along with experimental verification, is developed to describe the generation, propagation and reception of a Rayleigh wave using angle beam wedge transducers. The Rayleigh wave generation process using an angle beam wedge transducer is analyzed, and the actual Rayleigh wave sound source distributions are evaluated numerically. Based on the reciprocity theorem and considering the actual sound source, the Rayleigh wave beams are modeled using an area integral method. The leaky Rayleigh wave theory is introduced to investigate the reception of the Rayleigh wave using the angle beam wedge transducers, and the effects of the wave spreading in the wedge and transducer size are considered in the reception process. The effects of attenuations of the Rayleigh wave and leaky Rayleigh wave are discussed, and the received wave results with different sizes of receivers are compared. The experiments are conducted using two angle beam wedge transducers to measure the Rayleigh wave, and the measurement results are compared with the predictions using different theoretical models. It is shown that the proposed model which considers the wave spreading in both the sample and wedges can be used to interpret the measurements reasonably.

## 1. Introduction

Rayleigh waves produce elastic displacements and stress that are confined to the surface within a shallow depth of approximately one wavelength, so that they are suitable for detection and characterization of damages or material property changes confined to the surface or near surface of test components [1,2]. Compared with the bulk waves, they can propagate a long distance along the component surface with less beam spreading [3,4]. Rayleigh wave is generated and detected on the same side of the material, therefore, access to only one side of the material is required [5]. These advantages make Rayleigh wave a useful tool in ultrasonic nondestructive evaluation (NDE). 

Rayleigh waves can be generated by angle beam wedge transducers [6,7], comb transducers [8,9], interdigital transducers [10], etc.. Regarding an angle wedge transducer, a bulk longitudinal wave transducer is placed on a wedge of a low speed material with a certain angle. Because Rayleigh waves can be obtained efficiently with high energy, and the facilities are simple and cheap, angle wedge transducers are widely used in NDE with ultrasonic surface waves. 

Rayleigh waves are generally measured using angle beam wedge transducers [11], air-couple transducers [12] and laser interferometers [13]. The laser interferometer can be used to measure the absolute out-plane displacement of Rayleigh wave, but the detected surface needs to be strongly polished; moreover, the equipment is expensive. Although the air-coupled transducer can be used to receive Rayleigh wave in a non-contact method, the received results have low signal-to-noise-ratio because of high attenuation in air. Therefore, the angle beam wedge transducer is a good choice for receiving Rayleigh wave. 

The pitch-catch Rayleigh wave testing using two angle beam wedge transducers has been widely used for material characterization, such as surface roughness, acoustoelastic effects or residual stress, and microstructural changes [14,15,16]. These methods are based on the measurement of velocity change and dispersion or attenuation of Rayleigh waves. In order to improve the accuracy of Rayleigh wave detection, it is necessary to obtain accurate Rayleigh wave beams generated by the angle beam wedge transducers and to know the reception process of Rayleigh waves. Therefore, the waves propagate and spread in the wedge and on the sample surface should be analyzed precisely.

The propagation theory of Rayleigh waves has been widely studied since 1887, and a series of detailed works for calculation of the Rayleigh wave beams were provided by Achenbach and co-works [17,18,19,20], wherein elastodynamic reciprocity approach and Fourier transform approach were developed to model the Rayleigh wave beams. In their models, an ideal point load or line load is introduced as the sound source, so that such methods cannot be directly used for modeling the Rayleigh wave beams generated by an angle beam wedge transducer. A 3-D point source Rayleigh wave beam model for angle beam wedge transducers was proposed by Schmerr and co-works [21,22], in which, the calculated sound source for Rayleigh wave is used. This 3-D model improves the accuracy of Rayleigh wave beams of angle wedge beam transducers, but it is still an ideal model because the actual wedge shape is not considered. Recently, a further model for angle beam wedge transducer is developed with consideration of the wedge size, but without experiment verifications [6].

The reception process of Rayleigh waves affects the measurement results. When the propagating wave beams are known, the received wave amplitudes with different sizes of receivers will be considerably different, which is true for both bulk and Rayleigh waves [23,24,25]. The reception process of Rayleigh waves using the angle beam wedge transducers is complicated because the wave mode conversion and wave diffraction in the wedge need to be considered. However, there is no effective tool to model this reception process, and in most cases, the wave propagation and diffraction in the wedge are neglected. In some works, the on-axis wave magnitudes with a Gaussian-distributed line source are introduced as the received results [12,26,27]. Although this simple approach can provide some basic characteristics of the measurements and a data fitting method can make the measurements match the predictions, the impreciseness of this method is obvious, such as large fitted attenuation coefficients and different decreasing tendencies between the measurements and predictions in the near distance. Therefore, a detailed knowledge of the Rayleigh wave reception process and modeling method is essential for accurate NDE of Rayleigh waves using angle beam wedge transducers. 

This paper presents a model, supported by experimental measurements, that is used to accurately analyze the entire process of the generation, propagation and reception of Rayleigh wave using the angle wedge beam transducers. Section 2 presents the basic modeling method, wherein the area integral method is developed to model the Rayleigh wave beams, and a leaky Rayleigh wave theory is introduced to analyze the Rayleigh wave reception. In Section 3, the simulation results of the Rayleigh wave beams and received average results are provided and discussed; and they are compared with the predictions by some other model methods. In Section 4, the experiments are conducted. The comparisons of the theoretical and experimental results are shown, and detailed discussions on the measurements are provided. Section 5 is devoted to the conclusions of this study.

## 2. Theory 

**Figure 1:**
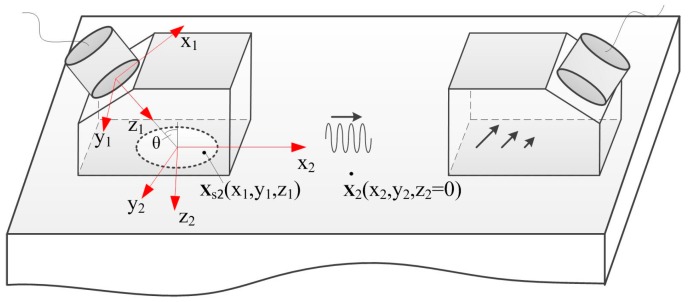
Schematic diagram of angle beam wedge transducers for generating and receiving Rayleigh wave.

We consider the case shown in Figure 1, in which, a contact transducer radiates longitudinal wave into a wedge, the Rayleigh wave is generated on the surface of a specimen when the longitudinal wave propagates to the specimen through the interface, and the propagating Rayleigh wave is received by another angle beam wedge transducer. The origin of the coordinate (x_1_y_1_z_1_) is in the center of transducer and that of (x_2_y_2_z_2_) locates in the intersection of transducer center axis and specimen surface, and the axis z_1_ and z_2_ are taken normal to the transducer and the plane stress-free surface of the specimen, respectively. In the following study, the specimen is considered as an isotropic elastic half-space solid. 

### 2.1. Rayleigh Wave Generation by an Angle Beam Wedge Transducer

**Figure 2:**
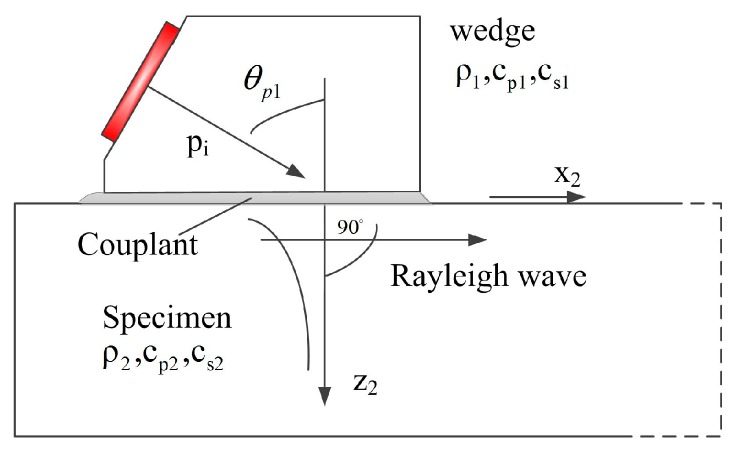
Generation of Rayleigh wave by an angle beam wedge transducer in the x-z plane.

We will focus on the Rayleigh wave generation process in the x-z plane at the solid-solid interface, as shown in Figure 2. When the bulk-wave transducer radiates a plane longitudinal wave into the wedge and this wave is obliquely incident on the smooth interface between the wedge and the specimen, there will be both longitudinal and shear waves transmitted. We write the incident longitudinal wave in a displacement potential form as [2]

(1) $$\varphi_{\mathrm{inc}1}=A_{i1}{\exp(\mathrm{ik}}_{p1}{(\mathrm{xsin}\theta }_{p1}+{\mathrm{zcos}\theta }_{p1})-i\omega t)$$

and the transmitted longitudinal and shear wave as:

(2) $$\varphi_{\mathrm{tp}2}=A_{t2}\exp\left[{\mathrm{ik}}_{p2}{(\mathrm{xsin}\theta }_{p2}+{\mathrm{zcos}\theta }_{p2})-i\omega t\right]$$

(3) $$\psi_{\mathrm{ts}2}=B_{t2}\exp\left[{\mathrm{ik}}_{s2}{(\mathrm{xsin}\theta }_{s2}+{\mathrm{zcos}\theta }_{s2})-i\omega t\right]$$

where $A_{i1}$, $A_{t2}$ and $B_{t2}$ are the amplitudes of potentials for incident longitudinal wave and transmitted longitudinal and shear waves, respectively, k=ω/c is the wave number, $\theta_{p1}$ is the incident angle, $\theta_{p2}$ and $\theta_{s2}$ are the transmitted angles for transmitted longitudinal and shear wave, respectively. The subscripts ‘1’ and ‘2’ denote that these parameters are for the wedge and specimen, respectively. 

When the incident angle is larger than the second critical angle, these transmitted waves are both inhomogeneous waves. At a certain incident angle, these longitudinal and shear waves will propagate on the surface of specimen as a Rayleigh wave. We introduce a Rayleigh wave number using Snell’s law:

(4) $$k_{r}=k_{p1}{\sin\theta }_{p1}=k_{p2}{\sin\theta }_{p2}=k_{s2}{\sin\theta }_{s2}$$

where $k_{r}{\;=\;\omega /c}_{r}$, and $c_{r}$ is the speed of the Rayleigh wave. Note, that in order to have an incident angle that satisfies Equation (4), it is necessary to have $c_{p1}<c_{r}$, i.e., the longitudinal wave speed of the wedge must be less than the Rayleigh wave speed of the specimen. Then the inhomogeneous longitudinal and shear waves can be written in terms of $k_{r}$ and $c_{r}$:

(5) $$\varphi_{\mathrm{tp}2}=A_{t2}\exp\left[-{\eta z}_{2}\left|k_{r}\right|\right]\exp\left[{\mathrm{ik}}_{r}{(x}_{2}-c_{r}t)\right]$$

(6) $$\psi_{\mathrm{ts}2}=B_{t2}\exp\left[-{\zeta z}_{2}\left|k_{r}\right|\right]\exp\left[{\mathrm{ik}}_{r}{(x}_{2}-c_{r}t)\right]\;$$

with $\eta \;=\;{\left(1-c_{r}^{2}/c_{p2}^{2}\right)}^{1/2}$ and $\zeta \;=\;{\left(1-c_{r}^{2}/c_{s2}^{2}\right)}^{1/2}$. It can be seen that these waves propagate mainly along the surface of the specimen and decay exponentially in the depth direction. 

Then we can get the expressions for x_2_- and z_2_- component displacements and stresses through the wave motion function of Equations (5) and (6), as follows:

(7) $$u_{x2}={[\mathrm{ik}}_{r}A_{t2}\exp(-{\eta k}_{r}z_{2})-{\zeta k}_{r}B_{t2}\exp(-{\zeta k}_{r}z_{2})]\exp\left[{\mathrm{ik}}_{r}{(x}_{2}-c_{r}t)\right]$$

(8) $$u_{z2}=[-{\eta k}_{r}A_{t2}\exp(-{\eta k}_{r}z_{2})-{\mathrm{ik}}_{r}B_{t2}\exp(-{\zeta k}_{r}z_{2})]\exp\left[{\mathrm{ik}}_{r}{(x}_{2}-c_{r}t)\right]$$

(9) $$\tau_{\mathrm{zz}2}=\mu_{2}\left[{(\zeta }^{2}k_{r}^{2}+k_{r}^{2}{)A}_{t2}\exp(-{\eta k}_{r}z_{2})+{2i\zeta k}_{r}^{2}B_{t2}\exp(-{\zeta k}_{r}z_{2})\right]\exp\left[{\mathrm{ik}}_{r}{(x}_{2}-c_{r}t)\right]$$

(10) $$\tau_{\mathrm{xz}2}=\mu_{2}\left[-{2i\eta k}_{r}^{2}A_{t2}\exp(-{\eta k}_{r}z_{2})+{(k}_{r}^{2}+\zeta^{2}k_{r}^{2}{)B}_{t2}\exp(-{\zeta k}_{r}z_{2})\right]\exp\left[{\mathrm{ik}}_{r}{(x}_{2}-c_{r}t)\right]$$

where $\mu_{2}$ is the Lamé constant of the specimen.

The boundary condition at the interface z_2_ = 0 of the specimen must satisfy:

(11) $$\tau_{\mathrm{zz}2}=\left(\zeta^{2}k_{r}^{2}+k_{r}^{2}\right)A_{t2}+{2\mathrm{ik}}_{r}^{2}{\zeta B}_{t2}=\{\begin{array}{ll} -p_{⊥1} & \text{under-wedge}\\ 0 & \text{stress-free} \end{array}$$

(12) $$\tau_{\mathrm{xz}2}=-{2i\eta k}_{r}^{2}A_{t2}+\left(\zeta^{2}+1\right)k_{r}^{2}B_{t2}=0$$

We can get the relationship between $A_{t2}$ and $B_{t2}$ from the stress-free boundary of Equation (11) as:

(13) $$A_{t2}=-\frac{2i\zeta }{\zeta^{2}+1}B_{t2}$$

and the stress-free boundary condition of Equation (11) requires that:

(14) $${\left(2-\frac{c_{r}^{2}}{c_{s2}^{2}}\right)}^{2}-4{\left(1-\frac{c_{r}^{2}}{c_{p2}^{2}}\right)}^{1/2}{\left(1-\frac{c_{r}^{2}}{c_{s2}^{2}}\right)}^{1/2}=0$$

which is the equation for the phase velocity of the Rayleigh wave.

We set arbitrary constant $B_{t2}=\;1$ and introduce the relationship between amplitudes of potential and displacement amplitudes to simplify the expressions of the Rayleigh wave displacements of Equations (7) and (8). We also introduce $u_{r}{(x}_{2}{,y}_{2},0)$ as the propagation Rayleigh wave beam on the surface of the specimen, so we can describe the displacement amplitudes of the Rayleigh waves in the three-dimensional coordinate as:

(15) $$u_{x2}{(x}_{2}{,y}_{2}{,z}_{2},t)=u_{r}{(x}_{2}{,y}_{2},0)[-\frac{2i\zeta }{\zeta^{2}+1}\exp(-{\eta k}_{r}z_{2})+i\zeta \exp(-{\zeta k}_{r}z_{2})]\exp\left[{\mathrm{ik}}_{r}{(x}_{2}-c_{r}t)\right]$$

(16) $$u_{z2}{(x}_{2}{,y}_{2}{,z}_{2},t)=u_{r}{(x}_{2}{,y}_{2},0)[\frac{2\eta \zeta }{\zeta^{2}+1}\exp(-{\eta k}_{r}z_{2})-\exp(-{\zeta k}_{r}z_{2})]\exp\left[{\mathrm{ik}}_{r}{(x}_{2}-c_{r}t)\right]$$

Equations (15) and (16) are the widely used expressions for describing the propagating Rayleigh waves on the surface. It can be seen that when these inhomogeneous waves reach the end of the wedge, they will satisfy the stress-free boundary conditions and continue to propagate as a Rayleigh wave. Therefore, Rayleigh waves can be efficiently generated by an angle beam wedge transducer if the wedge material and incident angle are chosen reasonably. Note that in the analysis process it is assumed only a longitudinal wave is radiated by the contact transducer, and reflected longitudinal and shear wave back to wedge are not listed. In addition, Rayleigh wave will re-radiate energy into the wedge (as a leaky Rayleigh wave) when it is generated underneath the wedge. However, here we just treat the propagating wave underneath the wedge as Rayleigh wave because the length of the wedge is usually very short and the wave speeds of Rayleigh wave and leaky Rayleigh wave are close, as we will analyze later. 

From the boundary condition at the interface of the specimen under the wedge one can find that the longitudinal wave sound pressures underneath the wedge will work as the sound source for generating Rayleigh wave [6]. Since the driving crystal of the contact transducer is in contact with the wedge through a couplant layer, it is reasonable to model this transducer as that of a uniform distributed source over the entire active area of its interface [2]. Based on the reciprocity theorem, the Sommerfeld-Rayleigh integral method can be used to obtain longitudinal wave pressures in the wedge as [2]:

(17) $$p_{1}{(x}_{1}{,y}_{1}{,z}_{1})=-{2\mathrm{ik}}_{p1}\int_{S_{T}}{\rho c}_{p1}v_{0}(x_{1}^{\prime },y_{1}^{\prime }{,0)G(x}_{1}{,y}_{1}{,z}_{1}|x_{1}^{\prime },y_{1}^{\prime },0){\mathrm{dS}}_{T}$$

where ρ is the density of the wedge, $v_{0}$ is the initial longitudinal wave particle velocity in the sound source, $S_{T}$ is the surface area of the bulk wave transducer, ${G(x}_{1}{,y}_{1}{,z}_{1}|x_{1}^{\prime },y_{1}^{\prime },0)\;=\;\frac{{\exp(\mathrm{ik}}_{p1}R_{1})}{{4\pi R}_{1}}$ is the Green’s function for 3-D wave motion equation [28], and $R_{1}\;=\;\sqrt{{{(x}_{1}-x_{1}^{\prime })}^{2}+{{(y}_{1}-y_{1}^{\prime })}^{2}+{{(z}_{1})}^{2}}$ is the distance from the sound source to the target point. Note that the wave attenuation in the wedge can be neglected because the propagation distance is short.

### 2.2. The Propagating Rayleigh Wave Beams

Rayleigh wave generation by incident longitudinal wave is analyzed and the actual Rayleigh sound source distributions can also be calculated numerically, but to get a better knowledge of the propagating Rayleigh wave beams of an angle beam wedge transducer, a Rayleigh wave model with the actual sound source is needed. The workaround for solving this problem is that we apply the reciprocity theorem to develop the integral representation theorem, derive fundamental solution of the wave motion equations using Green’s function method, and apply the solution into the actual condition [6].

**Figure 3:**
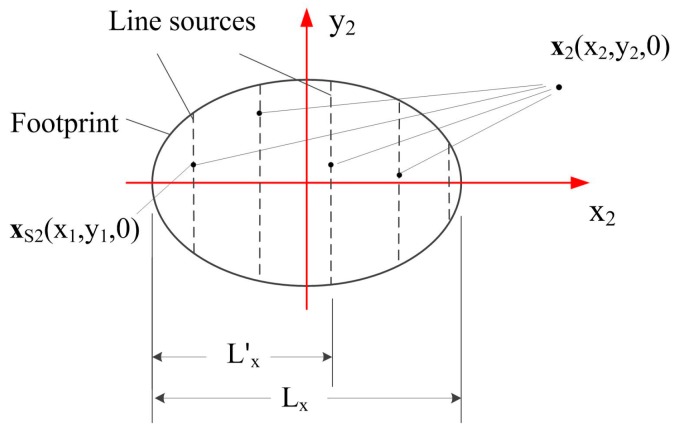
Geometry for calculating Rayleigh wave beams using an area sound source in the x-y plane.

As we mentioned above, when the Rayleigh wave beam $u_{r}{(x}_{2}{,y}_{2},0)$ on the surface of the specimen is known, the Rayleigh wave distributions in 3-D space can be obtained. Thus, we will first describe the method to model the Rayleigh wave beams in the x_2_-y_2_ plane. Figure 3 shows the case for modeling Rayleigh wave beams in the x_2_-y_2_ plane, in which, the active region of the Rayleigh sound source is in the so-called ‘footprint’ area which can be obtained using the exact geometry optics, when the sound beam in the wedge radiated by the contact transducer is assumed well collimating [29,30]. Since Rayleigh wave propagates in one direction only when an angle beam wedge transducer is used as the transmitter, we will model the Rayleigh wave beams in the forward propagating direction. We divide the area sound source into multiple banding sources which are perpendicular to the Rayleigh wave propagation direction and narrow enough to be treated as line sources. Considering one line source with the length of $L_{S}$, based on the reciprocity theorem, the Rayleigh wave beams generated by this line source can be expressed as the combination of the integral representation and Green’s function:

(18) $$u_{r}{(x}_{2}{,y}_{2},0)=\frac{-2}{c_{r}}\int_{L_{S}}v_{1}^{\prime }(x_{2}^{\prime },y_{2}^{\prime }{,0)G(x}_{2}{,y}_{2}|x_{2}^{\prime },x_{2}^{\prime }){\mathrm{dL}}_{S}$$

where ${G(x}_{2}{,y}_{2}|x_{2}^{\prime },y_{2}^{\prime })\;=\;\frac{-i}{4}\sqrt{\frac{2i}{k_{r}{\pi R}_{2}}}{\exp(\mathrm{ik}}_{r}R_{2})$ is the Green’s function for modeling the Rayleigh wave beams, $R_{2}\;=\;\sqrt{{{(x}_{2}-x_{2}^{\prime })}^{2}+{{(y}_{2}-y_{2}^{\prime })}^{2}}$, and $v_{1}^{\prime }(x_{2}^{\prime },y_{2}^{\prime },0)$ is the particle velocity of the Rayleigh sound source. 

Considering all the contributions from the actual sound source positions, the area sound source can be replaced by these line sources and the line integral formula from Equation (18) can be extended to the following form to express the surface integral as [6]:

(19) $$u_{r}{(x}_{2}{,y}_{2})=\frac{-2}{c_{r}L_{x}}\int_{S_{F}}v_{1}^{\prime }{(x}_{2}{,y}_{2}{)G(x}_{2}{,y}_{2}|x_{2}^{\prime },y_{2}^{\prime }{)\mathrm{dS}}_{F}$$

where $L_{x}$ is the equivalent length of the footprint area source, which is introduced to calculate the sound beams out of the footprint and given as:

(20) $$L_{x}={2a/\cos\theta }_{p1}$$

where a is the radius of the contact transducer. The integral area should cover the whole area underneath the wedge, although the sound sources out of the footprint have little effect on the Rayleigh wave beam distributions. Note that the equivalent length $L_{x}$ is introduced when the footprint is completely included underneath the wedge. 

Before we obtain the final integral expression for Rayleigh waves, we had to address the problem with the calculated longitudinal wave sound pressures that could not be employed directly as the Rayleigh sound source. In order to make sure that the pressures in the wedge are the same as those acting on the specimen from below at the interface, we introduce the following transmission coefficient [21]:

(21) $$T=\frac{\rho_{1}c_{p1}}{\rho_{2}c_{r2}}\left(T^{p;p}\frac{c_{s2}}{c_{p2}}\left(\frac{c_{p2}^{2}}{c_{s2}^{2}}-2\frac{c_{p2}^{2}}{c_{r2}^{2}}\right)-{2\mathrm{iT}}^{s;p}\frac{c_{s2}}{c_{r2}}\sqrt{\frac{c_{s2}^{2}}{c_{r2}^{2}}-1}\right)$$

where $\left(T^{p;p}{,T}^{s;p}\right)$ are ordinary plane wave transmission coefficients for the longitudinal and shear waves, respectively, for two elastic solids in smooth contact. 

Combining Equations (17) and (21) yields the Rayleigh sound source expression using particle velocity. Then, we place the source expression into Equation (19) and construct the expression for the propagating Rayleigh wave beams outside of the region underneath the wedge. We also introduce an attenuation coefficient $\alpha_{r}$ of Rayleigh wave to account the energy loss when the wave propagates on the surface, and get the final expression as:

(22) $$u_{r}{(x}_{2}{,y}_{2},0)=\frac{1}{L_{x}}\frac{k_{p1}v_{0}{(x}_{0}{,y}_{0}{,z}_{0})T}{{2\pi c}_{r}}\frac{\exp(i\pi /4)}{\sqrt{{2k}_{r}\pi }}\int_{S_{F}}\int_{S_{T}}\frac{{\exp(\mathrm{ik}}_{p1}R_{1})}{R_{1}}\frac{{\exp(\mathrm{ik}}_{r}R_{2}-\alpha_{r}R_{2})}{\sqrt{R_{2}}}{\mathrm{dS}}_{T}{\mathrm{dS}}_{F}$$

The Rayleigh wave beams can be obtained when the initial sound source over the transducer interface is used for the integrations. When the Rayleigh sound beam expression, Equation (22), is substituted into Equations (15) and (16), the Rayleigh wave distributions in 3-D space can be obtained. 

It is well known that, by the virtue of reciprocity, acoustic coupling between the incident longitudinal wave in the wedge and the Rayleigh wave on the surface of the solid is the same as that of the surface wave back into the wedge via leakage, therefore the wedge transducer has to be truncated before the end of the acoustic footprint to achieve maximum sensitivity. We should note that this model equation is also valid for this case. When the wedge partially covers the footprint, the actual sound source should be used, and the new equivalent length $L_{x}^{\prime }$ should also be selected [6]. 

### 2.3. Leaky Rayleigh Wave Theory and Reception of Rayleigh Wave

**Figure 4:**
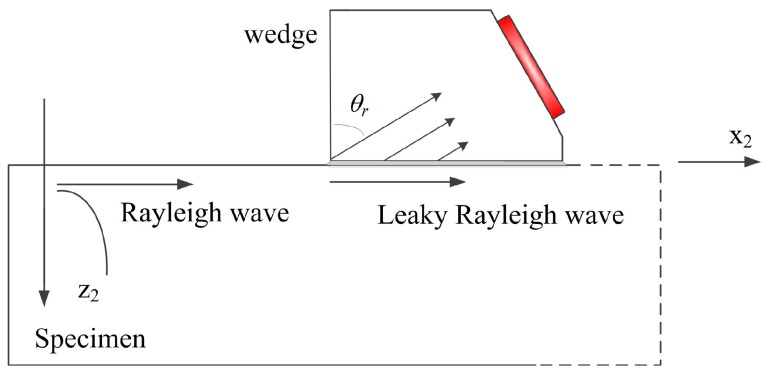
Sketch of Rayleigh wave reception by an angle beam wedge transducer, where (leaky) Rayleigh wave propagates on the specimen surface, and the decreasing length of the arrows in the wedge describes the amplitude diminishing of the leaky field under the wedge.

When another angle beam wedge transducer is introduced as the receiver, Rayleigh wave propagates along the specimen-wedge interface but will re-radiate wave energy into the contact wedge, and the reradiated wave is received by the bulk wave transducer. The coupled longitudinal wave re-radiated by Rayleigh wave is measured, and this process is described in Figure 4. In this case, a new boundary condition supporting wave propagation in the smooth contact solid-solid interface should be considered. In addition, the diffraction of the coupled wave propagating in the wedge should be considered; moreover, the effect of the receiver size on the received average wave results in the reception process should be taken into account. 

The theory of interface waves passing along a solid-solid interface has been researched in many studies [11,31]. There is a variety of interface wave types dependent on the parameters of the two solid materials and contact states (smooth contact or weld contact) between the two solids. In this study, the longitudinal wave speed in the wedge is lower than the Rayleigh wave speed observed on the specimen surface. Hence, the leaky Rayleigh wave and its theory can be introduced to investigate the reception process of Rayleigh wave [32]. 

In the boundary conditions of the smooth-contact wedge-specimen interface, the solution to the leaky Rayleigh wave speed will be a complex number, wherein the real part denotes the actual sound speed and the imaginary part implies the attenuation due to the leakage of energy [32,33,34]. In addition, the leaky Rayleigh wave speed $c_{\mathrm{Lr}}$ is slightly higher than the Rayleigh wave speed, i.e., $c_{\mathrm{Lr}}>c_{r}$. When Rayleigh wave propagates on the stress-free surface of the specimen and reaches the smooth contact wedge-specimen interface, it will propagate as the leaky Rayleigh wave and radiates energy into the wedge in the form of a coupled longitudinal wave at a leaky Rayleigh angle, as shown in Figure 4. The leaky angle $\theta_{\mathrm{Lr}}$ can be calculated using the Snell’s law as:

(23) $$\theta_{\mathrm{Lr}}=\sin^{-1}\left(c_{p1}/c_{\mathrm{Lr}}\right)$$

One should note that since the leaky Rayleigh wave speed is close to the Rayleigh wave speed, we have $\theta_{\mathrm{Lr}}\approx \theta_{p1}$, which indicates that the same angle beam wedge transducers can be used to generate and receive Rayleigh wave. In addition, the slight changes of the Rayleigh wave beams underneath the receiving wedge induced by the change of wave speed can be also neglected. 

As shown in Figure 4, the decreasing length of the arrows for describing the coupled longitudinal wave under the wedge due to the strong attenuation of leaky Rayleigh wave energy with increasing distance. In addition, when the coupled longitudinal wave propagates in the wedge, it will also spread. Therefore, the propagating longitudinal wave beam in the wedge is expressed as [2]:

(24) $$u_{p1}{(x}_{2}{,y}_{2}{,z}_{2})=-{2\mathrm{ik}}_{p1}\int_{S\prime }{\;T\prime \;u}_{r}(x_{2}^{\prime },y_{2}^{\prime },0)\exp(-\alpha_{\mathrm{Lr}}{l)G(x}_{2}{,y}_{2}{,z}_{2}|x_{2}^{\prime },y_{2}^{\prime },0)d\;S\prime$$

where ${G(x}_{2}{,y}_{2}{,z}_{2}|x_{2}^{\prime },y_{2}^{\prime },0)\;=\;\frac{{\exp(\mathrm{ik}}_{p1}R_{3})}{{4\pi R}_{3}}$ is the Green’s function which is the same as that for 3-D wave motion equations, $R_{3}=\sqrt{{\left(x_{2}^{\prime }-x_{2}\right)}^{2}+(y_{2}^{\prime }-y_{2}{)}^{2}}$, is the distance from the receiver surface to sound source, the integral area S′ denotes the whole area underneath the receiving wedge, T′ is an unknown reflected coefficient, and ${T\prime u}_{r}{(x}_{2}{,y}_{2},0)$ denotes the magnitude of sound source generating the radiated longitudinal wave in the wedge. The exponent term denotes the energy loss due to leakage, in which $\alpha_{\mathrm{Lr}}$ is the attenuation coefficient for leaky Rayleigh wave and l is the distance from the underneath source position to the front edge of the wedge. When the wave beam in the wedge is calculated, the ($x_{2}y_{2}z_{2}$) coordinate system for the Rayleigh wave beams is used. In addition, we do not need to consider the actual direction of these coupled longitudinal waves because the phases are included in the term $u_{r}{(x}_{2}{,y}_{2},0)$. 

These diffracted longitudinal waves are received by the contacted bulk wave transducer. However, the magnitudes of the received waves are affected by the receiver sizes. Therefore, considering the finite size of the receiver, the received average wave results are described by introducing another integral process as:

(25) $$\tilde{u}(x)=\frac{1}{S_{r}}\int_{S_{r}}\int_{S\prime }{T\prime u}_{r}(x_{2}^{\prime },y_{2}^{\prime },0)\exp(-\alpha_{\mathrm{Lr}}l){G(x}_{2}{,y}_{2}{,z}_{2}|x_{2}^{\prime },y_{2}^{\prime }{,0)\mathrm{dS}\prime \mathrm{dS}}_{r}$$

where $S_{r}$ denotes the active area of the receiver interface. Note that the sensitivity of the receiver interface is assumed to be equal. 

By substitution Equation (22) into Equation (25), the last expression for the received average wave results can be obtained. But in this study, we will focus on the distributions of the received results. So we can neglect these parameters which do not affect wave distributions, and obtain the further simplified expression as:

(26) $$\tilde{u}(x)∼\int_{S_{r}}\int_{S\prime }\int_{S_{F}}\int_{S_{T}}\frac{{\exp(\mathrm{ik}}_{p1}R_{1})}{R_{1}}\frac{{\exp(\mathrm{ik}}_{r}R_{2}-\alpha_{r}R_{2})}{\sqrt{R_{2}}}\frac{{\exp(\mathrm{ik}}_{p1}R_{3})}{R_{3}}\exp(-\alpha_{\mathrm{Lr}}{l)\mathrm{dS}}_{T}{\mathrm{dS}}_{F}{\mathrm{dS}\prime \mathrm{dS}}_{r}$$

We can obviously see from Equation (26) that when the wave frequency is known, the received results mainly depend on the transmitter and receiver sizes, the wedge sizes, Rayleigh wave attenuation and leaky Rayleigh wave attenuation. All these effects will be discussed in the following section. 

## 3. Simulation and Discussion

### 3.1. Rayleigh Sound Source under the Wedge

In this section, the longitudinal wave sound fields in the wedge radiated by a contact transducer are calculated. In the following simulations, the contact transducer radius a is 6.35 mm, the excitation frequency f is 2.25 MHz, and the initial uniform distributed sound velocity $v_{0}$ in the transducer face is assumed to be 1 m/s. The longitudinal wave propagates in a Lucite wedge, in which the longitudinal wave speed $c_{l}$ is 2725 m/s and the shear wave speed $c_{t}$ is 1120 m/s. The incident angle θ is 68° and the distance from the center of the transducer to the center of footprint is 40 mm. The length and width of wedge are about 28 mm and 33 mm, respectively. It is well known that the wedge will be truncated before the end of the acoustic footprint to achieve maximum sensitivity, so it is assumed that only half footprint is included in the underneath wedge. 

**Figure 5:**
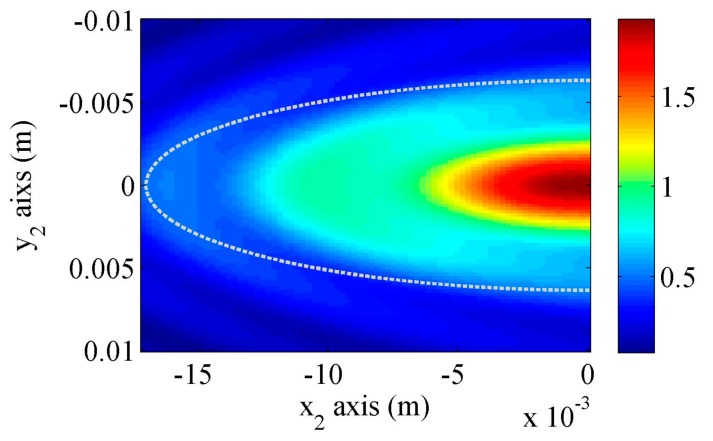
Longitudinal wave field distributions underneath the wedge generated by a uniform distributed contact transducer. Attenuation in the wedge is neglected.

Figure 5 shows the longitudinal wave sound field distribution under the wedge, in which the half-ellipse denotes the exact footprint. The wave fields are numerically calculated using Equation (17) in the (x_1_y_1_z_1_) coordinate. Note that there is a transformation of the coordinate systems, and the sound fields are plotted in the (x_2_y_2_z_2_) coordinate. It is observed that the main energy is concentrated in the footprint although there is energy out of this region because of the wave spreads. Since the wedge width in the y_2_ direction is much larger than the main beam width, the effects of vertical wall on the side wedge will be very small and can be neglected. This sound wave fields will be used as the Rayleigh sound source for calculating the Rayleigh wave beams in the following section. Also note that a line source with calculated or assumed distributions cannot be representative of the actual area sound source distributions, which will be discussed later. 

### 3.2. Rayleigh Wave Beam Distributions on the Stress-Free Surface

In this section, the Rayleigh wave beams on the aluminum specimen surface radiated by this angle beam wedge transducers will be simulated. The longitudinal, shear and corresponding Rayleigh wave speeds in aluminum are 6350 m/s, 3170 m/s and 2960 m/s, respectively. Two different attenuation coefficients of Rayleigh wave are used, i.e., $\alpha_{r}$ = 0 and 0.5 Np/m. The numerical calculated Rayleigh angle is very close to the incident angle in the wedge, so we can assume that the Rayleigh wave can be effectively generated. The Rayleigh wave beams are calculated using Equation (22) with the calculated Rayleigh wave sound source. Note that when half-footprint is covered by the wedge, as shown in Figure 5, the equivalent length $L_{x}^{\prime }$ has to be selected for the new values. 

**Figure 6:**
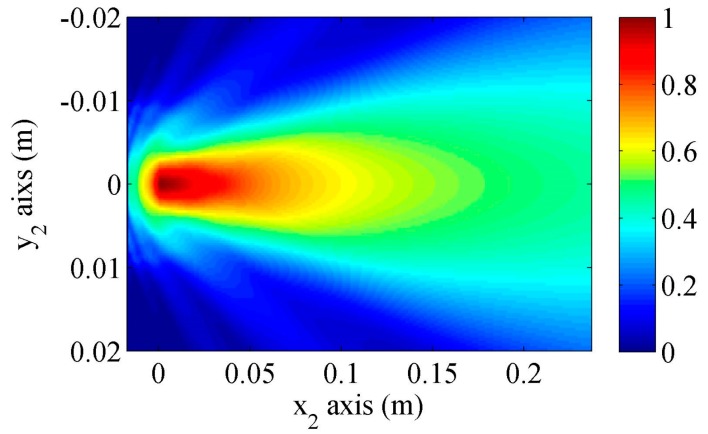
Rayleigh wave beam distributions on the surface predicted by the proposed model when the Rayleigh wave attenuation is neglected.

Figure 6 shows the fundamental Rayleigh wave beams calculated using the proposed model when the Rayleigh wave attenuation coefficient is set 0. Note that the Rayleigh wave beams under the wedge are also shown. The wave beam behaviors have been well discussed in the references [6,29].

**Figure 7:**
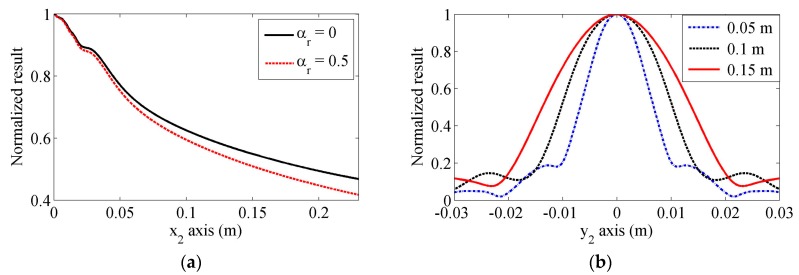
Normalized Rayleigh wave beam magnitudes along (**a**) x_2_-axis, and (**b**) y_2_-axis. Different Rayleigh wave attenuation coefficients are used in (**a**), but attenuation is neglected in (**b**).

Figure 7a shows the normalized magnitudes of the Rayleigh wave beams in the propagation direction. The decreasing tendency of the Rayleigh wave beams can be observed from the on-axis results, and the magnitude decreases more quickly when the attenuation is considered. The normalized results in the lateral direction at x_2_ = 0.05, 0.1 and 0.15 m, are shown in Figure 7b. It can be also seen from the lateral results that the main beam is located near the central axis, and the beam widths get larger as the propagation distance increases. In addition, the side lobes can be seen in this figure. These distributions show a basic behavior of the propagating Rayleigh wave beams, however, the received wave results obtained using another angle beam wedge transducer may be different from these results. In the following section, detailed comparisons between these results are presented. 

### 3.3. Received Wave Results by an Angle Beam Wedge Transducer

**Figure 8:**
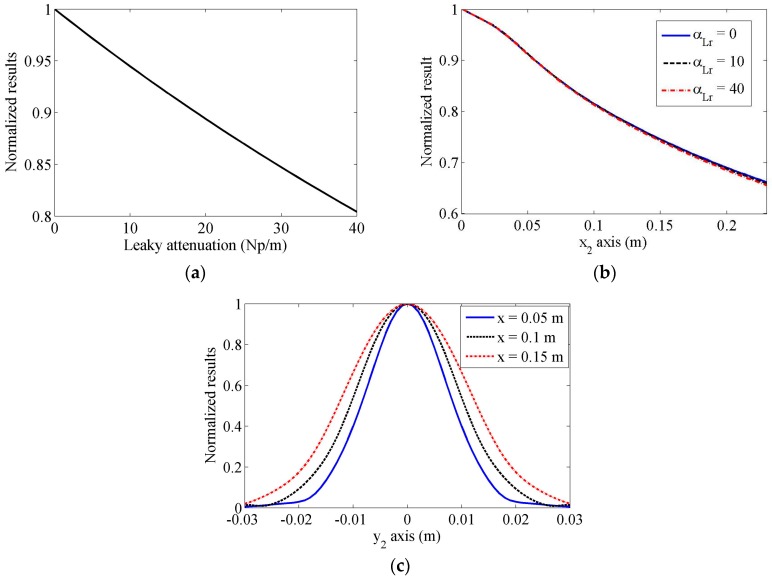
Distributions of normalized magnitudes of received wave results using an angle beam wedge transducer, (**a**) at the distance of x_2_ = 0.1 m, (**b**) along x_2_-axis, and (**c**) along y_2_-axis. Different leaky Rayleigh wave attenuation coefficients are used in (**a**,**b**), but attenuation is neglected in (**c**).

In this simulation, two same angle beam wedge transducers are used as the transmitter and receiver. Their parameters are the same as described above. Although it is well known that the leaky Rayleigh wave will attenuate severely, there is no exact attenuation coefficient to account for the lost energy numerically yet. Here, in order to investigate the effect of leaky Rayleigh wave attenuation, we show the normalized received average magnitudes in the propagation distance of x_2_ = 0.1 m, but using different leaky wave attenuation coefficient values. The results are shown in Figure 8a. It can be seen that the received magnitudes will be much smaller when a larger attenuation coefficient is used. The normalized received average magnitudes in different propagation distances using different leaky wave attenuation coefficient values ($\alpha_{\mathrm{Lr}}$ = 0, 10, 40 Np/m) are shown in Figure 8b, but we can find that there are no obvious differences between these normalized results. So we can draw that although the received magnitudes will be much smaller when a large leaky wave attenuation coefficient is used, the leakage does not affect the tendency of the received results in the propagation distance. Compared to the effect induced by the attenuation coefficient of Rayleigh wave, the influence of the attenuation coefficient of the leakage on the distributions of the received results is insignificant, though its value can be much larger than that of Rayleigh wave. Hence, in the following simulations, the attenuation factor for the leaky Rayleigh wave is neglected. If we compare the received results obtained using an angle beam wedge transducer and the on-axis beam distribution, one can clearly see that the decreasing tendencies are different. 

The normalized magnitudes of the received results in the lateral direction at propagation distances of x_2_ = 0.05, 0.1 and 0.15 m are indicated in Figure 8c. The results show that the maximum value exists at the central axis, and these magnitudes decrease quickly when the transducer moves away from the central axis. Compared with the lateral results shown in Figure 7b, it is found that the received magnitudes using the angle wedge transducer have very smooth behaviors, and the effects of side lobes are insignificant.

### 3.4. Received Wave Results Using Different Sizes of Receivers

**Figure 9:**
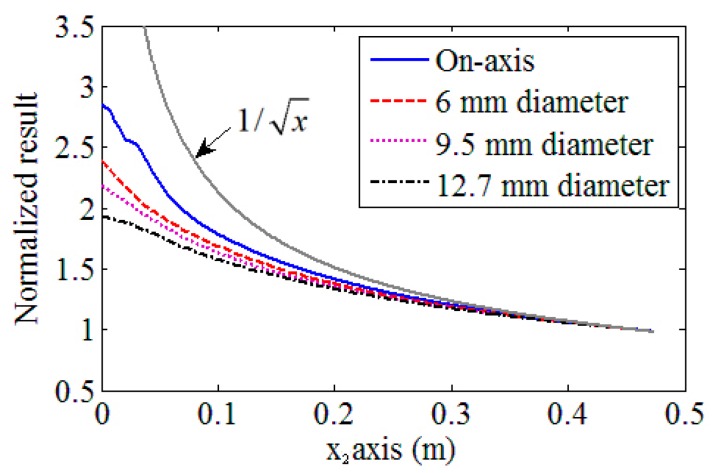
Tendencies of the received wave results using different sizes of receivers. Attenuations are neglected in the simulations.

We will discuss the effect of the receiver sizes on the received result distributions. In this simulation, three different sizes of the receiver elements (3, 4.75 and 6.35 mm radii) are used. The other parameters used here will be the same as mentioned previously. The attenuations are neglected as we focused on the magnitude tendency of the received results. Figure 9 shows the simulated results, in which, the on-axis wave beam results and received magnitudes using different sizes of angle beam wedge receivers are included and are normalized by their results for a distance of x_2_ = 0.45 m. It can be observed clearly from these results that at far distance, all these magnitudes will damp as follows: $\tilde{u}∼1/\sqrt{x}$, which is similar to that observed for cylindrical waves. However, in the near distance cases, these results have different damping tendencies, and a basic discipline is that the results obtained using a smaller size receiver will damp more quickly. These results indicate that the measured Rayleigh wave distributions will be influenced by the receiver size, and it is essential to consider the reception process in the practical measurement. 

### 3.5. Comparison Results with Other Models

In order to illustrate the importance of considering the beam spreading in the wedge and the receiver size when Rayleigh wave is receipted by an angle beam wedge transducer, the received results predicted by the proposed model are compared with the simulations using other simple methods. In the first simple model (Model 1), the wave propagation and spreading in the receiving wedge are neglected, so that the whole Rayleigh wave values underneath the receiving wedge are numerically added together to yield the received results. In the second model (Model 2), we introduce the on-axis results predicted using Equation (22) as the received results. Since the energy of Rayleigh waves is concentrated on the surface of the substrate and decays exponentially with depth, Rayleigh waves on a free surface can be expressed in a general form with a 2-D wave equation. A line source is usually used to model the Rayleigh wave beams and account for the beam spreading behaviors [2,17,35]. Therefore, in the third model (Model 3), the angle beam wedge transmitter is replaced by a Gaussian distributed line source, and the calculated on-axis results are used as the received results, which is described in the reference [26]. 

**Figure 10:**
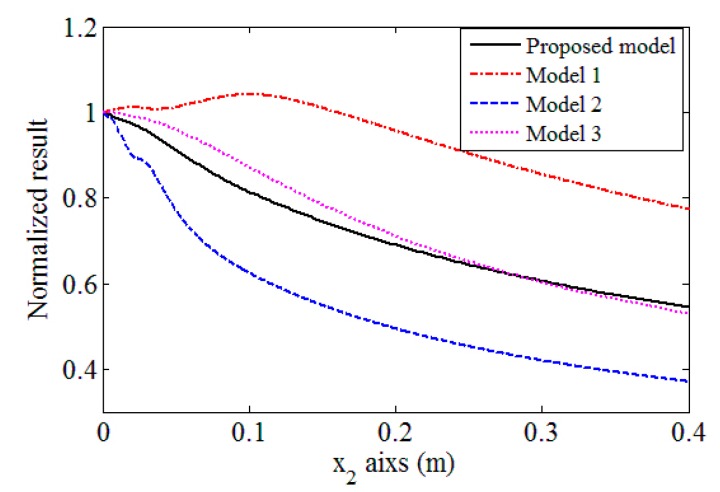
Received results along the propagation distance predicted using different models. Attenuations are neglected in the simulations.

The normalized simulations along the propagation distance by different models are plotted in Figure 10. The angle beam wedge transducer in Section 3.1 will be used as the transmitter and receiver. All the attenuations are neglected. Note that the element size of the receiver does not affect the simulation results of these simple models. When the Gaussian distributed line source is used, its half-length is assumed as the transducer radius. We can find that there are significant differences among these predictions. In general, the results in Model 1 have the lowest decreasing tendency and the predicted results will increase in the near propagation distance, while the results predicted by Model 2 decrease quickly in the near propagation distance. The results provided by Model 3 are similar to these predicted by the proposed model, however, the differences between them are obvious: in the propagation distance from 0.02 to 0.25 m, the results of Model 3 decrease slowly at first and then quickly; while the predictions of the proposed model show the opposite tendency. The different tendencies can cause some measured parameters for Rayleigh wave, such as attenuation, deviate from each other greatly, as will be shown in the following section. The experiments will be conducted to check the received results along the propagation distance and test the validities of these models later. 

## 4. Experiments and Results

**Figure 11:**
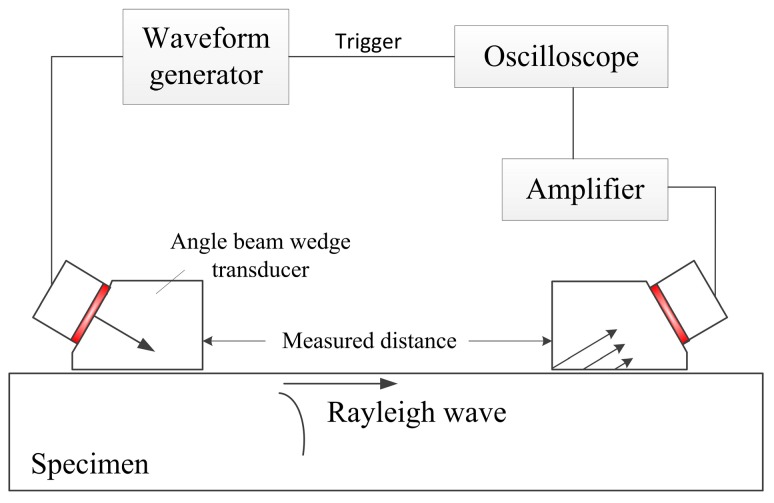
Sketch of experiment setup.

The experimental setup using the angle beam wedge transducers to generate and receive Rayleigh wave is depicted in Figure 11. A basic summary of the measurement follows here. The generating system is an acrylic wedge (ABWSL-1045, Olympus) coupled with an ultrasonic generating transducer (V404, Panametrics, Waltham, MA, USA, center frequency of 2.25 MHz and 6.35 mm radius), excited by an waveform generator (33611A, Keysight Technilogies, Inc., Santa Clara, CA, USA). The parameters of the angle beam wedge transducer are the same as those in the simulation section, and only half of the footprint is covered by the wedge. The input pulse shape was a 2.25 MHz sine wave modulated by a rectangular window of 30 cycles, so that Rayleigh wave with narrow band can be obtained. In the reception port, the angle beam wedge transducer is the same as the one in the transmission port. Rayleigh wave propagates on an Al-6061 surface and will be received by the receiver. The signal is amplified by a pulser-receiver (Panametrics-NDT 5072PR, Panametrics, Waltham, MA, USA) and digital using a Waverunner oscilloscope (TBS1154, Tektronix, Inc, Wilsonville, OR, USA). Light oil is used to couple the transducers to the wedges, as well as the wedges to the specimen. During the experiment process, the transmitter is fixed and the receiver is replaced when the propagation distance changes. 

### 4.1. Results in the Propagation Distance

Under the condition that two angle beam wedge transducers are aligned to each other, the received wave magnitudes in different propagation distances are measured first. A ruler is used to keep the two wedges aligned and determine the distance between two front wedge edges. The measured distances are from 0.02 to 0.2 m with the gap of 0.01 m, and the measurements will be repeated five times with all experiment setups kept in the same condition. Since the Specifications of the angle beam wedge transmitter and receiver are the same as those described in the simulation section, the measurements will be compared with the simulated results in Section 3. 

**Figure 12:**
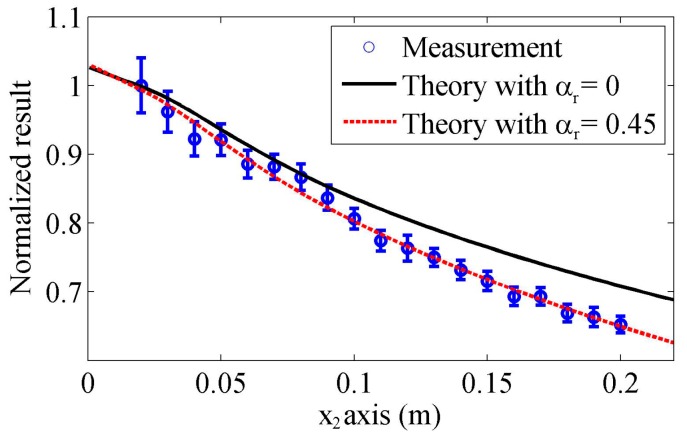
Measured normalized results in the propagation distance and comparisons with the predictions by our proposed model using different attenuation coefficients.

The normalized experiment results will be compared with the theoretical predictions using different models. Figure 12 compares the measurements and the predictions using our proposed model. The experiment results show the average and the standard deviation of five measurements. As we have analyzed in Section 3, only the attenuation of Rayleigh wave affects the distributions of the received wave results. Hence, in the predicted results, different Rayleigh wave attenuation coefficients are used: $\alpha_{r}=0$ and 0.45 Np/m. In the first case, the attenuation is neglected, while the second attenuation coefficient is extracted by fitting the predictions and average measured results. It can be observed that the measured results are in good agreement with the predictions when the attenuation is considered. The correlation coefficient between the predictions and measured average results is 0.98. Note that it is easy to distinguish the differences caused by attenuation and wedge geometry or receiver size in the simulated results, as in the propagation distance, the attenuation effect can be modeled using an exponential decay but the measured Rayleigh wave distribution behaviors are determined by the transducer geometry. Therefore, the agreement indicates the proposed model is effective in accounting for the wave spreading and receiver size. 

**Figure 13:**
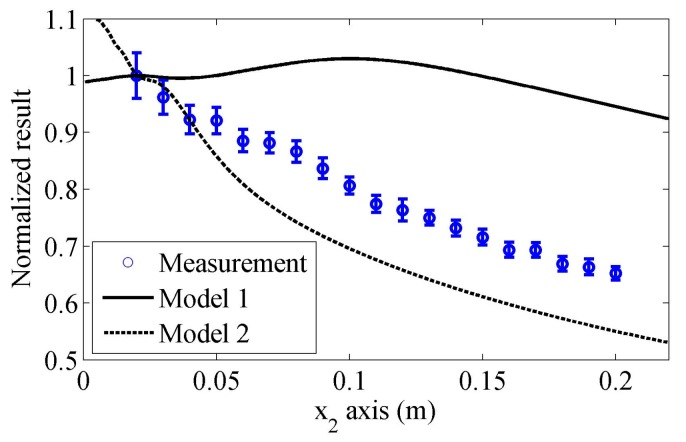
Measured normalized results in the propagation distance and comparisons with the predictions by Model 1 and Model 2.

Figure 13 shows the measurements and predictions using Model 1 and Model 2. One can observe that the results predicted by Model 1 are much different from the measurements, especially in the near distance range. We cannot obtain a good agreement between theoretical predictions and measurements even when a very large attenuation coefficient of Rayleigh wave is introduced. In addition, the predicted results using Model 2 without considering the attenuation will decrease more quickly than the measured results, thus, they cannot be used to predict the experiment results accurately. 

**Figure 14:**
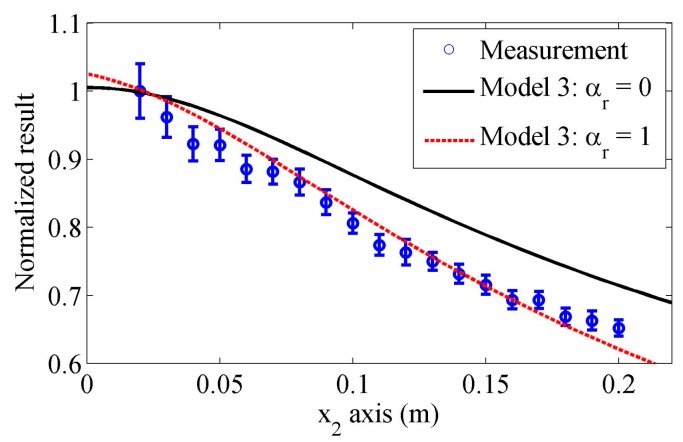
Measured normalized results in the propagation distance and comparisons with the predictions by the Model 3 using different attenuation coefficients.

The comparisons between predictions using Model 3 and measurements are shown in Figure 14. In order to make the theoretical calculations match with the experiment results, an attenuation coefficient of Rayleigh wave, $\alpha_{r}=1\;\mathrm{Np}/m$, is used. It is found that although the theoretical calculations will get close to the measurements, the differences between them in the decreasing tendencies are still significant. When a different size of receiver is used, the measurement results may have different behaviors, but this model cannot predict this change. Although when Model 3 is used one can match the theoretical predictions to measurements by adjusting the width of Gaussian source, it is hard to execute because different ranges of measurements are involved, one may obtain different width values. Thus, it has many limitations in the practical applications. 

It should be stressed that when the measurements and theoretical predictions are compared, the attenuation coefficients of Rayleigh wave are introduced. In order to distinguish the effects of the Rayleigh wave attenuation from these of beam spreading, the attenuation coefficients should be measured at first. However, accurate measurement of Rayleigh wave attenuation is still in study. It is shown that the attenuation of Rayleigh wave is very sensitive to surface roughness [36], and in some recent researches, the measured attenuation coefficients of Rayleigh wave at the polished surface of al6061 specimens vary from 0.6 to 2 Np/m at 2.25 MHz [26,27]. Part of reason for the variation of measured attenuations may be the differences of roughness and material composition. But in these measurements, Model 3 was used as the theoretical method to account for the wave spreading; through our analysis, this method is not so accurate and different attenuation coefficients can be extracted if the measurements in different propagation ranges are used, as shown in Figure 12. 

Since good agreement is observed between the predictions by our proposed model and the measurement results in the whole propagation range, it is more reasonable to think the extracted attenuation coefficient only accounts for dissipation. As an inverse problem, if the wave spreading on the surface and in the wedges is well considered, the attenuation of Rayleigh wave induced by the material dissipation can be measured accurately.

### 4.2. Results in the Lateral Distance

**Figure 15:**
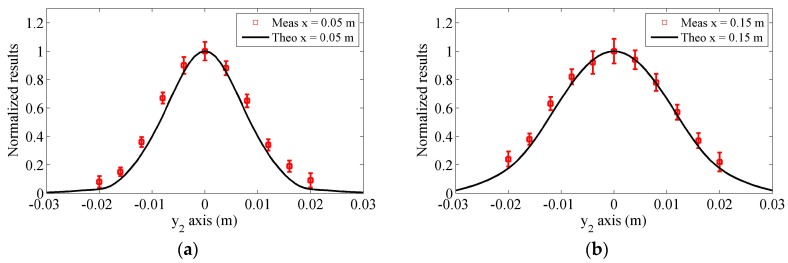
Measured normalized results in the lateral direction at the propagation distances (**a**) 0.05 m and (**b**) 0.15 m and comparisons with the predictions by our proposed model.

The received results in the lateral distance from −0.02 to 0.02 m will be measured at propagation distances of 0.05 and 0.15 m, respectively. The distance gap used in the measurements is selected as 4 mm. Figure 15a,b compares the experiment results with the predicted results obtained using the proposed model when the propagation distances are 0.05 and 0.15 m, respectively. In the theoretical predictions for each case, all of the attenuations are neglected because the wave propagates a same distance. It can be found that in the lateral position, the measured results will decrease smoothly, coinciding with the predicted results. In addition, because the main sound beams become wider in the longer propagation distance, the magnitudes of the results will decrease slowly in the lateral distance. Generally speaking, the proposed model can effectively predict measured lateral results. The tiny deviations between the predictions and measurements may be caused by the inaccuracy of the equipment and measurement errors. 

The deviations in these experiments are considerable, though the experiment conditions remain largely the same. The coupling state between the sample surface and the wedge, which is decided by the amount of coupling agent and clamping pressure, will affect the experiment results. In addition, the errors in the measurement distances and misalignment will affect the experiment results. However, in general, the predictions by the proposed model and experiment results show good agreement. Based on these comparisons, it can be concluded that the proposed model provides a more effective method than other simple models to consider the generation and reception process of Rayleigh waves using the angle beam wedge transducers as the transmitter and receiver. 

## 5. Conclusions

The generation, propagation and reception of Rayleigh waves using the angle beam wedge transducers employed as the transmitter and receiver are modeled in this study. The wedge size is considered and calculated sound wave distributions under the wedge are used as the Rayleigh wave source, thus, this model can be used to predict more accurate Rayleigh wave beams. In the reception process, the leaky Rayleigh wave theory is introduced and the wave spreading in the wedge is taken into account. It is shown that the distributions of the received wave results in different propagating distances are dependent on the receiver sizes and the attenuation of Rayleigh wave. The simulated results using the proposed model and three other simple models are compared, and the differences among these results are discussed in detail. The experiment measurements for Rayleigh wave on the surface of aluminum specimen helped in confirming the accurate predictions of the received wave distributions. 

The research provides an effective tool for Rayleigh wave nondestructive testing and evaluation using angle beam wedge transducers in practice. However, further works are still needed. In the derivation, the last results are obtained with four integrations, and it is shown that only a few parameters, such as transducer sizes, driving frequency and wave numbers affect the Rayleigh wave beam distributions. Therefore simplifying the calculation algorithms and defining diffraction corrections for the angle beam wedge transducers are necessary. In addition, when the leaky Rayleigh wave generates the coupled longitudinal wave in the solid-solid interface, the coupled coefficient should be derived. The measurement of absolute amplitudes or velocities of Rayleigh wave will be investigated.
